# Unmanned aerial vehicles: potential tools for use in zoonosis control

**DOI:** 10.1186/s40249-018-0430-7

**Published:** 2018-06-11

**Authors:** Qing Yu, Hui Liu, Ning Xiao

**Affiliations:** 1 National Institute of Parasitic Diseases, Chinese Center for Disease Control and Prevention; Chinese Center for Tropical Diseases Research; WHO Collaborating Centre for Tropical Diseases; National Center for International Research on Tropical Diseases, Ministry of Science and Technology, Key Laboratory of Parasite and Vector Biology, National Health Comission of China, 207 Ruijin Er Road, Shanghai, 200025 China; 2 Jinan Center for Disease Control and Prevention, Jinan, 250021 Shandong China

**Keywords:** Control, Echinococcosis, Public health, Unmanned aerial vehicles, Wild animal, Zoonosis

## Abstract

Unmanned aerial vehicles (UAVs) have become useful tools to extend human abilities and capacities. Currently UAVs are being used for the surveillance of environmental factors related to the transmission of infectious diseases. They have also been used for delivering therapeutic drugs and life-saving supplies to patients or isolated persons in extreme conditions. There have been very few applications of UAVs for disease surveillance, control and prevention to date. However, we foresee many uses for these machines in the fight against zoonotic disease. The control of zoonoses has been a big challenge as these diseases are naturally maintained in animal populations. Among 868 reported zoonoses, echinococcosis (hydatid disease) is one of the most severe public health problems and listed as one of 17 neglected tropical diseases targeted for control by the World Health Organization. Infected dogs (domestic or stray) play the most important role as definitive hosts in maintaining the transmission of echinococcosis. However, the actual contribution of wild canines to transmission has received little attention as yet, but should certainly not be ignored. This paper summarizes the history of development and application of UAVs, with an emphasis on their potential use for zoonosis control. As an example, we outline a pilot trial of echinococcosis control in the Qinghai-Tibet Plateau region, in which UAVs were used to deliver baits with praziquantel for wildlife deworming. The data suggested that this is a cost-effective and efficient approach to the control of zoonotic diseases transmitted among wild animal populations.

**Electronic supplementary material:**

The online version of this article (10.1186/s40249-018-0430-7) contains supplementary material, which is available to authorized users.

## Multilingual abstracts

Please see Additional file 1 for translations of the abstract into the five official working languages of the United Nations.

## Background

Technological progress and military demands have seen unmanned aerial vehicles (UAVs) gradually develop into an important component of weaponry worldwide since the 1950s. Meanwhile, many developed countries, such as the USA and Japan, have found civilian uses for UAVs, such as aerial spraying for plant crop protection. The first use of UAVs for pest control on cotton crops in the USA was in 1918 [1]. Since then, more than 20 types of UAVs for agricultural purposes have been produced in the USA and over 60% of pesticide sprayings are now done using these machines [2]. Since the 1990s, Japan has consistently been at the forefront of unmanned helicopter use for pest control. In 1990, the Japanese company Yamaha launched the first unmanned helicopter primarily for pesticide spraying [3–5]. Although development of UAVs in China was initiated in the 1950s for military purposes, civilian uses, primarily for agricultural spraying, have been expanding since 2008 [6–8]. Because they can operate under clouds and are light, relatively cheap, small and maneuverable, UAVs have been extensively applied in the fields of surveying and mapping, handling of emergency events, surveillance and recording of images [9–12]. They can provide very accurate data of land-use changes such as forest exploitation or agricultural development.

In the field of public health, UAVs have considerable potential. One of their main applications has been to acquire real-time data and constantly update important risk-related information in hotspot areas. The detailed ecological and environmental data they collect can be used for assessing factors (e.g. movement and distribution of people, animals, and pathogen-carrying insects) influencing the transmission of infectious diseases [13–16]. This is possible because spatial resolution by remote sensing of UAVs can reach the centimeter level. Given this imaging capability, UAVs can also be used for recovery and rescue activities in the aftermath of natural disasters, as for example, following the 2008 Sichuan and the 2010 Yushu earthquakes in China. On those occasions, they were mainly used in assessing the degree of damage to buildings and the environment [17]. In terms of disease surveillance and control/prevention, two main functions of UAVs are identification and action, and both are expected to be widely utilized in the future. In a survey on human malaria infection from monkeys, Fornace et al. (2014) used UAVs to delineate changes in habitats of mosquitoes and monkeys. Their data were integrated with case information from hospitals to assess the transmission risk [18].

A zoonosis is broadly defined as “a disease and/or an infection that is naturally transmitted between humans and vertebrates” [19, 20]. Table 1 lists some of the most important of these. Taylor et al. (2001) reviewed 1415 types (species) of infectious pathogens causing human disease, of which 868 (61%) were regarded as zoonotic [21]. Among these zoonotic diseases, echinococcosis (hydatid disease) is of major public health concern and one of the 17 neglected tropical diseases recognized by the World Health Organization. Echinococcosis occurs worldwide, but is mainly endemic in central, eastern and western Asia, South America, Australasia, southern, northern and eastern Africa. Echinococcosis is caused by tapeworms of the genus *Echinococcus*, primarily *E. granulosus* (which causes cystic echinococcosis, CE) and *Echinococcus multilocularis* (alveolar echinococcosis, AE). It is estimated that over 1 million people are at risk of CE and AE. Economic costs are around US$ 3 billion annually. The disease burden is nearly 10 million (due to CE) and 6.5 million (due to AE) disability-adjusted life years. AE is “tumor-like” and has a ten-year mortality rate of 94% if the patients are without sustained treatment [22, 23]. Dogs play a key role in maintaining transmission. In fact, through implementing control programs focused on deworming and management of domestic and farm dogs, New Zealand, Iceland and Tasmania have all successfully eliminated echinococcosis [24–26]. However, the contribution of wild canids to maintenance of zoonotic transmission has been neglected to date. Due to their wide-ranging behavior, the control of infected wild animals is still a big challenge. However, it is anticipated that UAVs could play a crucial role in control of zoonotic transmission with a focus on wild animal populations. Recent work is evaluating the use of UAVs to deliver praziquantel to deworm wildlife on the Qinghai-Tibet plateau as a means of controlling echinococcosis. This study highlights the extensive applications in public health that UAVs could have, particularly in surveillance and control of infectious diseases.

**Table 1:** Major zoonoses

Disease	Pathogen	Major reservoir host	Major intermediate host	Major transmission route
Angiostrongyliasis	*Angiostrongylus cantonensis*	Mice	Terrestrial and marine mollusks	Ingestion
Anisakiasis	*Anisakis* spp. and *Pseudoterranova* spp.	Marine mammals	Fish	Ingestion
Anthrax	*Bacillus anthracis*	Herbivores	-	Contact, inhalation, and ingestion
Brucellosis	*Brucella* spp*.*	Herbivores, pigs, and dogs	-	Contact
Campylobacter enteritis	*Campylobacter* spp*.*	Poultry and cattle	-	Ingestion
Clonorchiasis	*Clonorchis sinensis*	Humans, cats, dogs, mice, and other animals	Freshwater snails(first), freshwater fish(second)	Ingestion
Cryptosporidiosis	*Cryptosporidium parvum*	Humans, cattle, and other livestock	-	Ingestion
Diarrhea caused by enterohemorrhagic strains	Enterohemorrhagic *Escherichia coli*	Cattle and humans	-	Ingestion
Ebola-Marburg viral disease	Ebola virus and Marburg virus	Fruit bats	-	Contact
Hydatid disease	*Echinococcus* spp*.*	Canines	Herbivores and rodents	Ingestion
Ehrlichiosis	*Ehrlichia* spp*.*	Ruminants, rodents, and dogs	-	Tick bites
Variant Creutzfeldt-Jakob disease	Prion	Livestock	-	Ingestion
Giardiasis	*Giardia lamblia*	Humans, beavers, and other animals	-	Ingestion
Epidemic hemorrhagic fever	Hantaviruses	Rodents	-	Inhalation
Hepatitis E	Hepatitis E virus	Primates	-	Ingestion
Influenza	Influenza virus	Birds and humans	-	Inhalation
Black fever	Several species of *Leishmania* spp.	Humans and canines	-	Sandfly bites
Leptospirosis	*Leptospira interrogans*	Wild animals and livestock	-	Contact
Listeriosis	*Listeria monocytogenes*	Environment and animals	-	Ingestion
Lyme disease	*Borrelia burgdorferi*, *B. garinii*, and *B. afzelii*	Rodents	-	Tick bites
Melioidosis	*Burkholderia pseudomallei* and *Whitmore bacillus*	Animals and environment	-	Contact, ingestion, or inhalation
Paragonimiasis	*Paragonimus* spp.	Humans, dogs, cats, pigs, and carnivores	Freshwater snails (first), crustaceans (second)	Ingestion
Plague	*Yersinia pestis*	Rodents	-	Flea bites and inhalation
Psittacosis	*Chlamydia psittaci*	Birds	-	Inhalation
Q fever	*Coxiella burnetii*	Mammals, birds, and ticks	-	Inhalation
Rabies	Rabies virus	Canines	-	Biting or scratching
Salmonellosis	*Salmonella* spp.	Animals	-	Ingestion
Schistosomiasis	*Schistosoma* spp.	Animals	Snails	Contact with contaminated water
Taeniasis	*Taenia solium* and *T. saginata*	Humans	Cattle and pigs	Ingestion
Tetanus	*Clostridium tetani*	Animals	-	Wound contact
Toxoplasmosis	*Toxoplasma gondii*	Cats and other felines	Sheep, goats, rodents, pigs, cattle, chickens, and birds	Ingestion
Trichinellosis	*Trichinella* spp.	Animals	-	Ingestion
Tularemia	*Francisella tularensis*	Rodents	-	Arthropod bites, contact, ingestion, and inhalation
Endemic typhus	*Rickettsia typhi*	Small rodents such as mice	-	Contact with flea feces
Yersiniosis	*Yersinia enterocolitica* and *Y. pseudotuberculosis*	Animals	-	Ingestion and contact
Skin fungal disease	*Microsporum* spp. and *Trichophyton* spp.	Animals	-	Contact
Hookworm disease	Three species of *Ancylostoma* spp. and *Necator americanus*	Humans, cats, dogs	-	Contact
Tuberculosis	*Mycobacterium tuberculosis*, *M. africanum,* and *M. bovis*	Mammals such as humans and cattle	-	Inhalation and ingestion
Japanese encephalitis B	Japanese encephalitis virus	Mosquitos, mammals, and birds	-	Mosquito bites

## Potential and challenges for UAV use in control of zoonoses

The prevention and control of zoonoses involves a complicated ecological network including the pathogens, human beings, livestock, wild animals and environmental factors [19]. When these natural circulations are affecting public health and threatening human lives, they often exhibit the following characteristics:

1. Huge resource requirements. Many zoonotic diseases impose large demands on public health resources and have high maintenance costs, requiring long-term and persistent measures and investment to maintain control. If control efforts stop, these diseases will rebound very quickly.

2. Emerging infectious disease issues. About 75% of emerging infectious diseases are zoonoses [21]. Their multi-host features provide potential pathogenic variations due to gene mutations and new pathogens may arise as a consequence [27]. For example, the avian influenza A virus H7N9 subtype that infects people resulted from the recombination of viruses in poultry [27, 28]. In addition, the overlap between new production activity areas of humans and the ecological niches of animals already living there promotes the dissemination of certain animal diseases to humans [29, 30].

3. Food safety issues. Many food-borne diseases are due to zoonotic pathogens that threaten human health. In 2011, 3.6 million people suffered from food-borne bacterial diseases in the USA [31], as did 330 000 people in Europe [32].

Many zoonoses are endemic in China. Among them, echinococcosis is one of the most severe public health problems in the western parts of China where the number of people at risk and the prevalence are the highest in the world. Eight provinces (autonomous regions) of China experience the greatest incidence of echinococcosis, Inner Mongolia, Sichuan, Yunnan, Tibet, Gansu, Qinghai, Ningxia, and Xinjiang. Domestic and farm dogs form the most important reservoir. A national survey in 2012 found 4.26% of dogs were positive for echinococcosis based on the copro-antigen test [33, 34]. The infection rate in stray dogs was higher than that in owned dogs and in some areas, such as Yushu of Qinghai Province, even reached 70% [35]. *Echinococcus* adult worms begin egg production in the small intestine of dogs 28–45 days after hydatid cysts have been eaten. For that reason, it is recommended to deworm every dog monthly. In 2006, the Chinese government initiated a national echinococcosis control program for “monthly drug-deworming of every owned dog”. This measure has achieved considerable success [36]. However, infection rates in wild canids (foxes and wolves) are poorly known but are thought to be significant. Efficient control of wild animal infections will be required if prevention and control efforts are to succeed [24, 37]. A pilot trial using UAVs for drug-bait delivery has been conducted in the field, which aimed to control echinococcosis in wild animals in highly endemic environments. In this trial, the parallel uses of bait delivery by UAVs and by manual methods in 1 km^2^ test areas were compared with respect to time of delivery and overall cost. Based on coproantigen tests, the average infection rate of wild animals was 38.2% higher in manually seeded areas than in those seeded by the UAVs, and only a third of the manpower was needed for the latter approach. In addition, the mean cost of a UAV to distribute baits was 40% of the cost of manual delivery. Furthermore, estimates based on a unit of one km^2^ area (equal to one million m^2^) indicate that the use of UAVs for distributing baits would cost approximately 61% less than manual delivery [38]. This highlights the potential applications of UAVs as efficient and cost-effective tools for zoonosis control in wild animals.

Recently, the Japanese Avionics company developed an infrared thermal-imaging camera with 320 × 240-pixel image dimensions and a 0.04 °C temperature resolution [39]. Therefore, it may be possible to apply UAVs for temperature monitoring in the surveillance of disease symptoms. Similarly, with the high accuracy and non-invasive nature of UAVs, this technology could be used to directly observe the activities and densities of rodent populations and to monitor changes in their mortality patterns, providing an early warning for natural focal diseases such as plague. With their increasing technical sophistication, UAVs also have the potential to perform population surveillance of mosquitoes through the delivery and recovery of mosquito-lure lamps. If the optical resolution eventually reaches an appropriate scale, it could be used for direct observation and monitoring of mosquitoes. If appropriate modifications are made to existing models used for environmental surveillance and pesticide spraying, UAVs could easily be used for control of disease vectors/intermediate hosts and for environmental disinfection. For example, UAVs could perform accurate fixed-point delivery of therapeutic drugs to treat animal infectious sources. They could also be used for targeted delivery of those vaccines that can be ingested or inhaled by animal populations [40–42]. In the event of disease outbreak or other disaster, UAVs could deliver therapeutic drugs and life-saving supplies to patients or isolated persons in remote areas.

## Conclusions

Although there have been very few applications of UAVs for disease surveillance and control to date, progress made in other fields leads us to expect that UAVs will be very useful tools for public health fieldwork, especially for the control of zoonotic diseases transmitted among wild animal populations.

## Additional file

Additional file 1: Multilingual abstracts in the five official working languages of the United Nations. (PDF 212 kb)

## Abbreviations

AE: Alveolar echinococcosis
CE: Cystic echinococcosis
UAVs: Unmanned aerial vehicles
