# Supplementary material for: Unmanned aerial vehicles: potential tools for use in zoonosis control
Source: Infect Dis Poverty. 2018 Jun 11;7:49. doi: 10.1186/s40249-018-0430-7 (PMC5994646; doi:10.1186/s40249-018-0430-7)
Supplement: Supplementary file 1 — Multilingual abstracts in the five official working languages of the United Nations. (PDF 212 kb) [file 40249_2018_430_MOESM1_ESM.pdf]

Translation of the abstract into the five official working languages of the United Nations

**الطائرات بدون طيار: وسائل محتملة للاستخدام في مكافحة الأمراض ذات أصل حيواني**

تشينغ يو وهوي ليو ونينغ شياو

**الملخص**
أصبحت الطائرات بدون طيار (UAVs) وسائل مفيدة لتوسيع القدرات والمهارات البشرية. تستخدم الطائرات بدون طيار حالياً لمراقبة العوامل البيئية المتعلقة بانتقال الأمراض المعدية . كما تم استخدامها أيضاً لتسليم الأدوية العلاجية والإمدادات المنقذة للحياة للمرضى أو الأشخاص المعزولين في الظروف القاسية. هناك عدد قليل جدًا من تطبيقات الطائرات بدون طيار لمراقبة الأمراض والسيطرة عليها ومنعها حتى الآن. ومع ذلك، نتوقع استخدامات كثيرة لهذه الآلات في مكافحة الأمراض ذات أصل حيواني. لقد كان التحكم في الأمراض ذات أصل حيواني تحديًا كبيرًا لأن هذه الأمراض تستمر بشكل طبيعي في مجموعات الحيوانات. ومن بين 868 من الأمراض ذات أصل حيواني التي تم الإبلاغ عنها، يعد داء المشوكات (مرض الأكياس المائية) واحدًا من أشد مشاكل الصحة العامة وأُدرج كأحد 17 من الأمراض المدارية المهملة التي تهدف منظمة الصحة العالمية إلى التحكم بها. تلعب الكلاب المصابة (المنزلية أو الضالة) الدور الأكثر أهمية كمضيفات حاسمة في استمرار انتقال مرض المشوكات. ومع ذلك، فإن الإسهام الفعلي للكلاب البرية في الانتقال لم يحظ باهتمام كبير حتى الآن، ولكن بالتأكيد لا ينبغي تجاهله. تلخص هذه الورقة تاريخ تطور وتطبيق الطائرات بدون طيار، مع التركيز على استخدامها المحتمل للتحكم في الأمراض ذات أصل حيواني. وكمثال على ذلك، فإننا نوجز تجربة رائدة للتحكم بمكافحة المشوكات في منطقة هضبة تشينغهاي - التبت، حيث استخدمت طائرات بدون طيار لتوصيل طعوم البرازيكوانتيل للتخلص من الديدان البرية. وتشير البيانات إلى أن هذه الطريقة فعالة من حيث التكلفة وفعالة لمكافحة الأمراض الحيوانية التي تنتقل بين مجموعات الحيوانات البرية.

Translated from English version into Arabic by Free Bird and Abdessalam AIT, through

TRANSLATORS
WITHOUT BORDERS

**无人飞行器：具有应用前景的人兽共患病预防控制工具**

余晴，刘辉，肖宁*

无人飞行器已经成为扩展人类能力的有效工具，它现在已用于与传染性疾病传播有关的环境因素监测，也常常用于极端条件下病人治疗药物的投送和绝境时的生命救援。时至今日，尽管在疾病预防控制与传播因素监测上，应用无人飞行器的报道并不多见，但是可以预见无人飞行器在人兽共患病预防控制方面将会有广阔的应用前景。由于人兽共患病拥有能保持自然传播的庞大动物群体，因此人兽共患病的防治面临着严峻的挑战。棘球蚴病是目前已经报道的 868 种人兽共患病的一种，也是世界卫生组织认定的 17 种严重危害公共卫生安全的被忽视热带病之一。尽管感染的家犬或流浪犬是传播此病的终末宿主并起着至关重要的作用，然而野生犬科类传染源在维持传播中所起的作用，至今没有引起足够的关注，但是很显然是不能忽视的。本文在总结无人飞行器发展历程的基础上，重点讨论了无人飞行器在人兽共患病预防与控制方面的潜在应用价值。同时，本文以青藏高原地区棘球蚴病防治过程中，针对野外犬科类传染源采用无人飞行器投放含有吡喹酮驱虫药饵的探索性试验为例，提出使用无人飞行器投放驱虫药饵的方式，对于在野生动物中开展人兽共患病的防治，是一项经济且有效

的措施。

Translated from English version into Chinese by Qing Yu and Ning Xiao

**Drones aériens (véhicules aériens sans équipages): Éventuels recours dans le contrôle des zoonose**

Qing Yu, Hui Liu et Ning Xiao

**Résumé**

Les drones aériens (véhicules aériens sans équipage) (UAVs) sont devenus des outils nécessaires pour étendre les aptitudes et les capacités humaines. Les drones aériens sont actuellement utilisés dans la surveillance des facteurs environnementaux liés à la transmission des maladies infectieuses. TIls ont aussi été utilisés pour la livraison de médicaments thérapeutiques et de kits de secours au bénéfice de patients ou de personnes isolées dans des conditions extrêmes. Jusqu'à présent, les drones aériens ont été très peu utilisés dans la surveillance, la lutte et la prévention contre les maladies. Toutefois, ces moyens aériens nous permettent d'envisager de nombreuses possibilités dans la lutte contre les maladies zoonotiques. La lutte contre les maladies zoonotiques a été un important défi puisque ces infections sont naturellement présentes dans les populations animales. Parmi les 868 cas de zoonoses signalés, l'échinococcosis (maladie hydatide) est l'une des infections les plus sévères en matière de santé publique et est classée comme l'une des 17 maladies tropicales négligée faisant l'objet d'un grand suivi par l'Organisation mondiale de la santé. Les chiens infectés (domestiques ou errants), considérés comme des hôtes définitifs, jouent le rôle le plus important dans le maintien de la transmission de l'échinoccosis. Cependant, la participation effective des canidés sauvages dans la transmission de la maladie n'a pas encore fait l'objet d'une attention suffisante mais elle ne devrait pas être ignorée. Ce document résume l'histoire et l'usage des drones aériens et met l'accent sur leur potentielle utilisation dans la lutte contre les zoonoses. A titre d'exemple, nous présentons un essai pilote de lutte contre l'échinococcosis dans la région du plateau de Qinghai au Tibet, région dans laquelle des drones aériens ont été utilisés pour lancer des appâts au praziquantel, dans le but d'effectuer un traitement vermifuge de la faune sauvage. Toutes les données nous mènent à croire que ce moyen est une solution économique et efficace dans la lutte contre les maladies zoonotiques transmises parmi les populations d'animaux sauvages.

Translated from English version into French by Vero marie and Windja, through

TRANSLATORS
WITHOUT BORDERS

**Беспилотные летательные аппараты: потенциальный инструмент для использования при надзоре за зоонозными инфекциями**

Цин Юй, Хуэй Лю и Нин Сяо

**Аннотация**
Беспилотные летательные аппараты (БПЛА) стали полезным инструментом, расширяющим человеческие возможности и способности. В настоящее время БПЛА используются при наблюдении за факторами окружающей среды, которые связаны с передачей инфекционных заболеваний. Также они применяются для доставки лекарственных средств и предназначенных для спасения жизни предметов пациентам либо лицам, изолированным в экстремальных условиях. По состоянию на данный момент БПЛА крайне редко применялись с целью контроля, надзора и профилактики заболеваний. Однако мы прогнозируем многочисленные виды использования указанных аппаратов в борьбе с зоонозными заболеваниями. Надзор за зоонозными инфекциями представляет собой серьёзную проблему вследствие того, что указанные заболевания естественным образом поддерживаются среди популяции животных. Из 868 зарегистрированных зоонозных инфекций довольно серьёзную проблему для общественного здравоохранения представляет эхинококкоз (гидатидная болезнь), который входит в список 17 игнорируемых тропических болезней, намеченных для контроля Всемирной организацией здравоохранения. В поддержании передачи эхинококкоза наиболее существенную роль выполняют заражённые собаки (как домашние, так и беспризорные), которые являются окончательным хозяином. Однако несмотря на важность проблемы, по состоянию на текущий момент не было уделено должного внимания непосредственному участию диких псовых в передаче таких инфекций. В настоящей работе обобщается история развития и применения БПЛА с акцентом на их возможное использование в надзоре за зоонозными инфекциями. В качестве примера приводится экспериментальное исследование, проведенное в рамках надзора за эхинококкозом в регионе Цинхай-Тибетского нагорья. В этом исследовании БПЛА задействовались для доставки приманок с празиквантелом с целью дегельминтизации диких животных. Полученные данные свидетельствуют об экономичности и эффективности указанного подхода в надзоре за передачей зоонозных заболеваний среди популяций диких животных.

Translated from English version into Russian by Liudmila Tomanek and Ann Nosova, through

TRANSLATORS
WITHOUT BORDERS

## Vehículos aéreos no tripulados: posibles herramientas para el control de las zoonosis

Qing Yu, Hui Liu y Ning Xiao

**Resumen**
Losvehículos aéreos no tripulados (UAVs, por sus siglas en inglés) se han convertido en herramientas útiles para aumentar las habilidades y capacidades de los humanos. Actualmente, los UAVs se usan para vigilar factores ambientales relacionados con la transmisión de enfermedades

infecciosas. También se han usado para entregar fármacos y suministros médicos que salvan vidas a pacientes o personas aisladas en condiciones extremas. Hasta la fecha, los UAVs se han usado en muy pocas ocasiones para vigilar, controlar y prevenir enfermedades. Sin embargo, prevemos muchos usos para estas máquinas en la lucha contra las enfermedades zoonóticas. El control de las zoonosis ha sido un gran desafío, ya que estas enfermedades se mantienen de forma natural dentro de las poblaciones animales. Dentro de las 868 zoonosis descritas, la equinococosis (hidatidosis) representa uno de los problemas más graves de salud pública y figura como una de las 17 enfermedades tropicales desatendidas objeto de control de la Organización Mundial de la Salud. Los perros infectados (ya sean domésticos o callejeros) desempeñan el papel más importante en la transmisión de la equinococosis como hospedadores finales. Sin embargo, hasta ahora, la contribución real de los cánidos salvajes a la transmisión ha recibido poca atención, pero sin duda debería ser tomada en cuenta. Este artículo resume la historia del desarrollo y aplicación de los UAVs y hace hincapié en su posible uso para el control de las zoonosis. Como ejemplo, describimos una prueba piloto de control de equinococosis en la región de la meseta del Tíbet, donde se usaron UAVs para entregar cebos con praziquantel para desparasitar a la fauna salvaje. Los datos parecen indicar que se trata de un enfoque económico y eficiente para el control de las zoonosis transmitidas entre poblaciones de animales salvajes.

Translated from English version into Spanish by Camila Kohen and Noelia Bernárdez, through

TRANSLATORS
WITHOUT BORDERS
